# Yi Liu answers questions about 15 years of research on covalent organic frameworks

**DOI:** 10.1038/s41467-020-19301-y

**Published:** 2020-11-16

## Abstract

Dr. Yi Liu is the facility director at the Molecular Foundry, Lawrence Berkeley National Laboratory. Trained as a supramolecular chemist and after a postdoctoral stay and working on click chemistry in Sharpless’s group he started his independent research career working on organic electronics, porous materials, and covalent organic frameworks (COFs). His aim is to develop nanostructured electronic materials through the design, synthesis and manipulation of tailor-made molecular constituents.

Tell us a little bit about your research background and how you became interested in the chemistry of porous materials and COFs.

I was trained as a supramolecular chemist in Fraser Stoddart’s group at UCLA during my graduate studies. Back then I used covalent and non-covalent bonds to organize electron donors and acceptors. My postdoc was about using click chemistry to make super-strong adhesive polymers in Barry Sharpless’s group. This group is known for pioneering very traditional organic chemistry. Later the group developed a different perspective on organic reactions in which they use very simple but highly efficient chemistry to make useful materials. I think that is a very important perspective. I developed a very strong interest in organic electronics since my graduate school and started working in this area when I moved to Berkeley lab. I became interested in COFs and in porous materials in general because I feel this is a reticular platform where charge carriers can be manipulated via the proper choice of building blocks and chemistry. I worked a lot with linear conjugated polymers. COFs emerge as an ordered, higher dimensional polymeric system which also have a very defined structure control. Particularly 2D COFs are reminiscent of other 2D materials such as graphene and transition metal dichalcogenides, where structure anisotropy plays a fundamental role. That is where I got interested and got into the field. The fact that the area is built off the concept of dynamic covalent chemistry is another reason why I am interested in COFs, as dynamic covalent chemistry represents the forefront of supramolecular chemistry and is one of my affections.

Looking back on 15 years research on COFs, what were the highlights in COF research and which expectations remained unfulfilled?

First of all, I think it has been a wonderful 15 years for COFs. Starting as a subfield of metal organic frameworks (MOFs) it now grows bigger and bigger and so does the impact. There are more excellent contributions than I can think of, but I will start with the seminal work 15 years ago on the original boronate ester COFs and later on the imine COFs. My personal favorite would be the growth of COF single crystals and the unambiguous structure identification by X-ray studies. That work is very challenging but was elegantly executed. The importance is apparent to me as a milestone in terms of confirming the extended high ordered COF structure. Before that, the closest was to use micro electron diffraction, which is a TEM technique, to resolve and model the structure but I think the single crystal work is particularly very remarkable.

COFs are a class of light weight, ordered, porous organic polymeric material. Many reported functional COFs have taken advantage of the porous properties. There is greater expectation though, for COFs to outperform what is already out there, especially the closely related MOF family. I am quite excited about COFs being depicted as a conceptual framework that can guide the flow of mass, ions or charges, and I remain highly interested in seeing the development of semiconducting COFs and their integration in optoelectronic devices. That may bring new aspects to the table, complementary to what has been demonstrated by linear conjugated polymers. Such expectation however still remains unfulfilled.

In your opinion, which challenges need to be tackled in order to move the field further?

The first one came to my mind is the control of crystallinity. With few exceptions, the majority of COFs are semicrystalline powders. It is easier to see the crystalline part, but the amorphous components are usually hidden. In other words, COFs are often obtained as imperfect polycrystals with uncontrolled defects and tiny crystalline domains, which may be overwhelming and limit the material’s properties. The semicrystalline nature also poses a characterization challenge. XRD is currently the primary characterization method but it is not capable of providing localized information in smaller domains. On the other side it is laborious to use TEM and other more localized methods to characterize such a system. Characterization has been a challenge and the solution may lie in the discovery of new and improved chemistry to increase crystallinity. Still we only have around a dozen of reliable chemical reactions to make COFs, which dictates crystallinity and properties. Breakthrough in controlling the thermodynamic synthesis, either by new reactions or creative means to control the crystallization process, may bring us closer to better control the structural integrity, analogous to how carbon–carbon bond formation can be biased toward diamond production under the rightful conditions.

Another challenge for certain applications is the poor solution processability. COFs are 2D or 3D polymers with periodic structures. In essence, they are crosslinked polymers, which imply that they typically have low solubility and are incompatible with solution processes. Depending on the applications, we need a clever way to deposit COFs on substrates or interfaces to overcome the processability barrier, which is not a problem for the linear polymer counterparts which have much better solution processability. This is especially important when it comes to optoelectronic applications.

What needs to be done in order to make COFs more widely adopted and to get a foothold in industry and industrial applications?

This is an important question to ask. I do feel the field fits more within the academic venue at the current stage. An upfront issue is the cost. Compared to traditional polymers or even to the closely related MOFs, the COF monomers are more like specialty chemicals. Chemists are able to design and make any monomer, but at the end the availability can be an issue. Another problem is upscaling. Most of the lab scale COF synthesis happens on the milligram scale, which is completely reasonable and fine for discovery purpose, though there is a missing link between the lab scale and the larger scale of a batch production. It also appears that the hydrothermal synthesis is quite sensitive to small changes of reaction conditions, which adds additional concerns for upscaling production or industrial applications.

Another point I want to make is, that in order to advance COFs toward industry we still need to demonstrate a unique application of COFs which outperforms other materials candidates.

Where do you see the field going next?

I think it is all related to what we have discussed before. I am looking at it in two parts: structure wise we still need to find better ways to grow large area and more crystalline and less defective COFs, ideally with facile access to single or few layer structures. This might benefit from advances in other areas, in particular from advances in synthesis such as high throughput synthesis with automation or potentially autonomous synthesis. This might help to accelerate the discovery and to improve the structure integrity. The development of artificial intelligence and machine-learning algorithms might help with this process, if coupled with high throughput synthesis.

Function wise, energy storage is one of the areas that COFs will continue to contribute to. This area would benefit from new carbon based porous materials as components in batteries or capacitors, electrodes, membrane separators, or solid state electrolytes. In my view these are territories where COFs might find a unique position. It is also potentially a greener materials source in terms of the element availability and environmental footprint.

In terms of optoelectronics, much more needs to be demonstrated in order to find applications in this field. Personally, I like to see a high mobility COF based transistor or a COF based organic photovoltaic with a reasonable efficiency. These are areas where we see great advances based on linear conjugated polymers, and COF presumably can be a strong competitor if we find the right building blocks and smart ways to improve processability. That would be a big step toward application of COF materials in microelectronics or nanoelectroncis.

*The interview was conducted by Senior Editor Dr. Johannes Kreutzer.*

